# LNC00673 suppresses proliferation and metastasis of pancreatic cancer via target miR-504/ HNF1A

**DOI:** 10.7150/jca.32855

**Published:** 2020-01-01

**Authors:** Yi Gong, Hai-su Dai, Jun-jie Shu, Wei Liu, Ping Bie, Lei-da Zhang

**Affiliations:** Department of Hepatobiliary Surgery, Southwest Hospital, Army Medical University, Chongqing, 400038, China.

**Keywords:** LINC00673, MiR-504/HNF1A, Proliferation, Metastasis, Pancreatic Cancer

## Abstract

Pancreatic cancer is a highly invasive malignant tumor of the digestive system. To explore the mechanism of pancreatic cancer development, development, invasion and metastasis, in this study we focused on long non-coding RNA (LncRNA), which has been reported to be involved in tumorigenesis. We identified a LINC00673, which is highly correlated with the pancreatic cancer risk. LINC00673 Overexpression is associated with good survival in pancreatic cancer patients, Effects of LINC00673 on pancreatic cancer cell apoptosis, viability, migration. LINC00673 negatively correlated with miR-504 and MiR-504 overexpression promoted cancer progression in Pancreatic cancer. MiR-504 negatively correlated with HNF1A, which was highly expressed Pancreatic cancer. HNF1A inhibited cell progression in pancreatic cancer cells. LINC00673 overexpression inhibited caner progression in nude mice. Taken together, LINC00673 can through suppress miR-504/ HNF1A regulating invasion and migration in pancreatic cancer. Also, we identified miR-504 as a target of LINC00673 in pancreatic cancer and LINC00673 can be used as a novel therapeutic target for the pancreatic cancer.

## Introduction

Pancreatic cancer is a highly invasive type of malignant tumor with a very poor prognosis 1. The 5-year survival rate is less than 4%, and only 10%-20% of patients have surgical indications 2. Progress in the diagnosis and treatment of pancreatic cancer depends on elucidation of the pathophysiological mechanisms involved, and it is important to study the mechanisms of pancreatic cancer development, invasion and metastasis. However, the underlying mechanisms leading to pancreatic cancer are unclear.

LncRNA is a non-coding RNA molecule longer than 200 nucleotides 3. LncRNAs involved in tumor cell proliferation, apoptosis, invasion and metastasis^4^. However, they do not encode proteins^4^.

Previous studies have reported that LINC00673 can promote epithelial-mesenchymal transition (EMT), proliferation and metastasis in thyroid carcinoma 5. Overexpression of LINC00673 promotes invasion and metastasis which associated with a poor prognosis in tongue squamous cell carcinoma 6. Lin et al. showed that the rs7214041 gene site of LINC00673 is highly correlated with the incidence of pancreatic cancer 7. LINC00673 plays an important role in maintaining cellular homeostasis, and its germline variability affects pancreatic cancer susceptibility.

Our previous study showed that downregulation of LINC00673 promotes PDAC cell proliferation by inhibiting HNF1A. Here, we describe a functional role of LINC00673 in suppressing the regulation of invasion and migration by miR-504. Additionally, we identified miR-504 as a target of LINC00673 in pancreatic cancer. Our findings will help develop potential therapies for the treatment of pancreatic cancer metastasis.

## Materials and methods

### Cell lines

A human Pancreatic duct epithelial cells line (HPDE), HEK-293T and three human pancreatic cancer cell lines (CanPan-1, Hs-766T, CFPAC-1) were obtained from Chinese typical culture preservation center. All cell lines were cultured in RPMI 1640 medium (Gibco) with 10% fetal bovine serum, at 37°C and 5% CO_2_.

### miRNA, SiRNA and plasmid transfection

The sequences of siRNA were listed in Table 1. SiRNAs of LINC00673, LINC00673, miRNA mimics and inhibitors were designed and synthesized by Ruibo Guangzhou. Transfection methods are described as references 8.

### Western blotting

After transfection, the cells were collected and lysed. Western blot analysis methods are as described in the references^9^. Antibody dilution ratio is as follows Cleaved-caspase-3 (1:1000, CST, #9579), HNF1A (1:1500, abcam, #ab204306), GAPDH (1:5000, CST, #51332).

### RNA extraction and quantitative real-time PCR

RNA was extracted from cells or tissues using TRIzol reagent (Invitrogen, Carlsbad, CA) and subjected to qRT-PCR. LINC00673, miR-504 and RNU6 expression was tested by qRT-PCR using the primer set from RiboBio (Guangzhou, China), the method as reference^10^. The primer sequences information was defined as in Table 1.

### RNA pull-down test

Biotinylated LINC00673 was obtained from Sangon Biotech, Shanghai, which was transfected into the cells with cellular protein extract (1 mg) for 24 hours. Then the cells were disrupted and centrifuged, and the supernatant collected. Dynabeads® M-280 Streptavidin (BD Biosciences) was applied to absorb biotinylated miR-504 along with LINC00673. And then, the product is washed and dehydrated for qRT-PCR detection.

### CCK-8 proliferation assay

After transfection, evaluation of cell proliferation using the CCK-8 assay (promega) 8.

### Flow cytometric analysis

Apoptosis assay was performed using FITC Annexin V Apoptosis Detection Kit I (BD Biosciences) according to the manufacturer's protocol. Stained cells were analyzed by FACSCalibur Flow Cytometer (BD Biosciences)^9^.

### Transwell assay

After processed, cells were stained with a 0.4% crystal violet solution. The invading cells were imaged using a digital microscopy (Nikon) 10. The assay uses 8.0μm Transwell Permeable Supports (Corning).

### Luciferase reporter assay

Experiments were performed in triplicate. Luciferase activity was measured using the dual-luciferase assay system (Promega, Madison, WI). The 293T cells were co-transfected with pmirGLO-LINC00673-WT, pmirGLO-LINC00673-MUT, pmirGLO-miR-504-3'UTRWT or pmirGLO-miR-504-3'UTR-MUT reporter plasmids and mimics NC, miR-504 mimics accordingly. After 48 hours of incubation, cells were subjected to a luciferase reporter assay^11^, ^12^.

### Animal experiments

Animal experiments were conducted using six-week-old female athymic (nu/nu) mice. Canpan-1 cells stably transfected with a series of lentiviruses were constructed in our laboratory. Next 8 nude mouse were divided into each groups and were injected with 4×10^6^/100µl cells. The mice were sacrificed 15 days after injection after tumor volume reached 1500 mm^313^.

### Immunohistochemistry (IHC)

xenograft specimens IHC assay were performed as described previously 8. The IHC staining score was reviewed by an expert panel of pathologists. human rabbit Ki-67 antibody (1:400; CST, #9027). The secondary antibody working solution kit containing DAB (Maxim Biotech, Inc.) The slides were reviewed using a light microscope (magnification, x100).

### The tissue sources

Cytoplasmic staining was brownish yellow for protein positive expression. According to the staining intensity of positive cells and the distribution range of positive cells, there are 4 grades: no cell staining (-); 1%-30% of cells are weak and moderately positive (+); 30% of cells are strongly positive or 30 %-60% of cells with moderate positive reaction were (++); more than 60% of cells with strong positive reaction were judged as (+++).

### Statistical analyses

Statistical significance was analyzed by unpaired Student's t tests or one-way ANOVA and Duncan's multiple range tests using the SAS statistical software package version 6.12 (SAS Institute) and p values less than 0.05 were considered statistically significant; Kaplan-Meier survival analysis was used to calculate the overall survival rate of pancreatic cancer^14^.

## Results

### Overexpression of LINC00673 is corrected with good prognosis in pancreatic cancer patients

**Figure 1:**
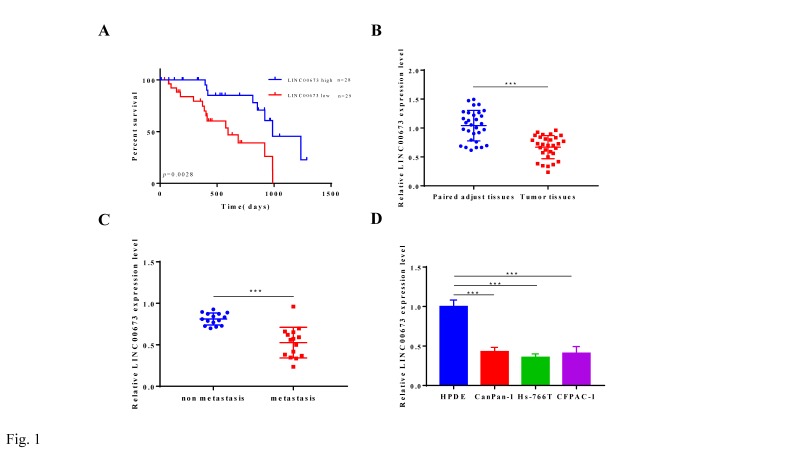
LINC00673 is associated with good survival in pancreatic cancer patients. (A) Association of LINC00673 expression with overall survival of pancreatic cancer patients (B) Relative expressions of LINC00673 in 30 pairs pancreatic cancer tissues normal ones. (C) Relative expressions of LINC00673 in pancreatic cancer tissues metastasis and non-metastasis. (D) Relative expressions of LINC00673 in pancreatic cancer cell lines and normal cell lines. *P<0.05, *** P<0.001.

The data obtained in this study demonstrated that lower LINC00673 RNA levels in pancreatic cancer patients were correlated with poor overall survival outcomes in Fig. 1A, which implied that LINC00673 acts as a potential suppressor oncogene and presents powerful prognostic value for pancreatic cancer patients. Our data showed that LINC00673 was significantly decreased in primary tumor samples from pancreatic cancer patients compared with adjacent tissues (Fig. 1B). In addition, LINC00673 expression was significantly decreased in specimens from pancreatic cancer patient with metastasis compare to non-metastasis (Fig. 1C). Our data also demonstrated that the expression of LINC00673 was significantly decreased in pancreatic cancer cell lines compared to human pancreatic duct epithelial cells (HPDE) (Fig. 1D).

### LINC00673 suppress pancreatic cancer cell grow and metastasis

**Figure 2:**
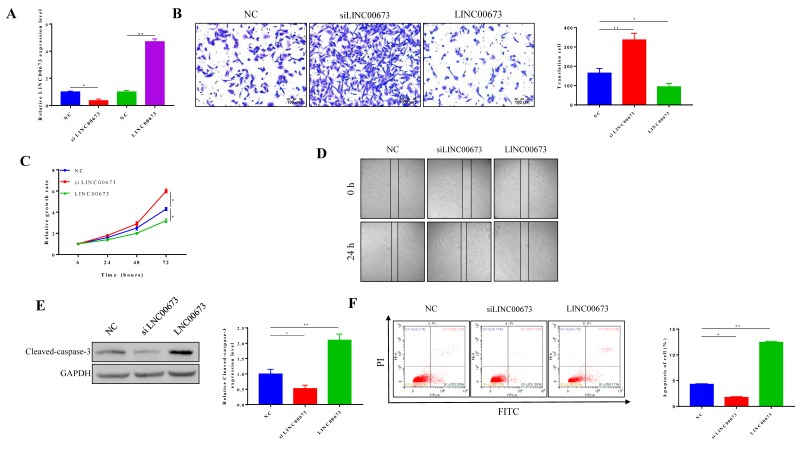
Effects of LINC00673 on pancreatic cancer cell apoptosis, viability, migration. (A)Validation of LINC00673 overexpression or knockdown in CanPan-1 cells as determined by qRT-PCR. (B) Transwell invasion assay of LINC00673 over-expression or knock-down in CanPan-1 cells. (C) CCK-8 proliferation assay of LINC00673 over-expression or knock-down in CanPan-1 cells. (D)Expression Cleaved-Caspase 3 in LINC00673 over-expression or knock-down in CanPan-1 cells as determined by western blot. (E)Flow cytometric analysis of apoptosis in LINC00673 over-expression or knock-down in CanPan-1 cells. *P<0.05, ** P<0.01

To investigate the function of LINC00673 in the tumorigenesis of pancreatic cancer, we used CanPan-1 cell lines, which exhibit intermediate expression of LINC00673. We transfected siRNA specific to LINC00673 in CanPan-1 cells and detected LINC00673 expression at 72 h post transfection (Fig. 2A). When LINC00673 was overexpressed, a lower migration rate was detected, but higher migration rates were observed when LINC00673 was knocked down (Fig. 2B). At the same time, CCK-8 assays showed that cell viability was significantly reduced in LINC00673-overexpressing cells (Fig. 2C). The cell apoptosis assay further supported the above inference. Less cell death appeared in the siLINC00673 group, whereas more cell death occurred in the LINC00673-overexpressing group, as shown by Western blotting (Fig. 2D) and flow cytometry (Fig. 2E).

### MiR-504 is a target of LINC00673

**Figure 3:**
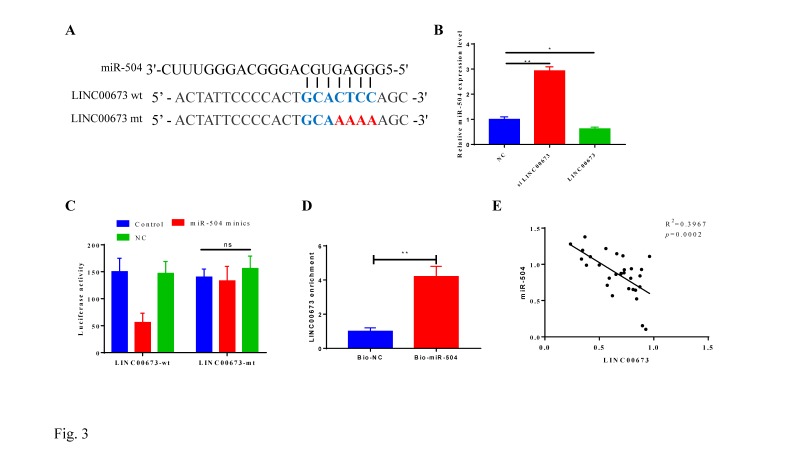
LINC00673 negatively correlated with miR-504. (A) The predicted binding sites of miR-504 to the LINC00673 sequence. (B) Expression of miR-504 LINC00673 over-expression or knock-down in CanPan-1 cells as determined by qRT-PCR. (C) Luciferase reporter assay of miR-504 directly targeted at lncRNA-LINC00673. (D)RNA pull-down test of miR-504 could pull down LINC00673. (E) Negative correlation between LINC00673 and miR-504 expression in pancreatic cancer patients. *P<0.05, *** P<0.01

A direct target relationship between LINC00673 and miR-504 was predicted by StarBase v2.0 (Fig. 3A). We transfected LINC00673 or siLINC00673 into CanPan-1 cells and detected the miR-504 expression level 72 h post transfection, and we found that the miR-504 expression increased when LINC00673 was disturbed (Fig. 3B). A luciferase reporter assay showed significantly lower luciferase activity in the miR-504 mimic+LINC00673-wt group than in the miR-504 mimic+LINC00673-mut group (Fig. 3C). Similarly, greater LINC00673 enrichment was observed in the bio-miR-504 group, indicating a strong correlation (Fig. 3D). In addition, linear regression analysis demonstrated that LINC00673 was negatively correlated with miR-504 after qRT-PCR detection of 30 tissue specimens (Fig. 3E).

### MiR-504 overexpression promoted cancer progression in Pancreatic cancer

**Figure 4:**
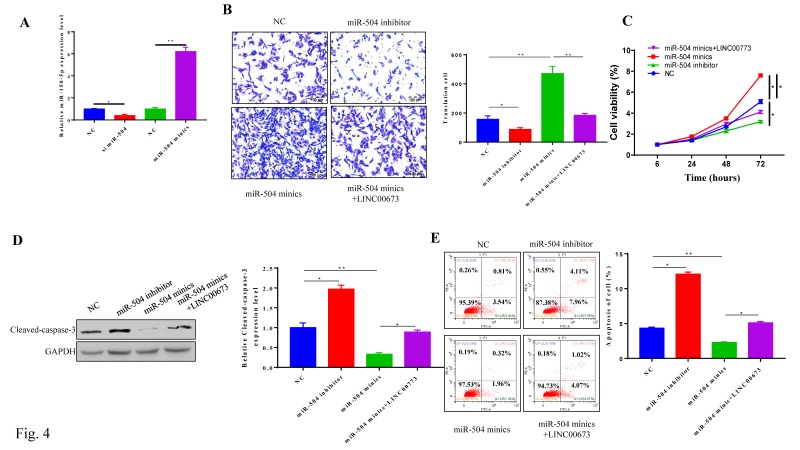
MiR-504 overexpression promoted cancer progression in Pancreatic cancer. (A)Validation of miR-504 mimics or inhibitor in CanPan-1 cells as determined by qRT-PCR. (B) Transwell invasion assay in miR-504 mimics\inhibitors and LINC00673 0verexpression transfected CanPan-1 cells. (C) CCK-8 proliferation assay of miR-504 minics\inhibitors and LINC00673 0verexpression transfected CanPan-1cells. (D) Expression Cleaved-Caspase3 in miR-504 mimics/inhibitors and LINC00673 overexpression transfected CanPan-1cells as determined by western blot. (E)Flow cytometric analysis of apoptosis in miR-504 mimics/inhibitors and LINC00673 overexpression transfected CanPan-1cells. *P<0.05, ** P<0.01

We next transfected inhibitors or mimics specific for miR-504 into CanPan-1 cells and detected miR-504 expression levels 72 h post transfection (Fig. 4A). The miR-504 inhibitor suppressed cell migration in the miR-504 inhibitor group but promoted migration in the miR-504 mimic group, and these effects were reversed by LINC00673 (Fig. 4B). Furthermore, miR-504 overexpression promoted pancreatic cancer cell proliferation, and this process was reversed by LINC00673 (Fig. 4C). The cell apoptosis rate was lower when miR-504 was up-regulated but higher when miR-504 was inhibited (Fig. 4E). Consistent with the apoptosis analysis, miR-504 overexpression induced the expression of the pro-apoptotic protein cleaved-caspase3, but LINC00673 reversed this effect (Fig. 4D). Together, our data suggest that miR-504 overexpression promoted cancer progression in pancreatic cancer.

### MiR-504 negatively correlated with HNF1A

**Figure 5:**
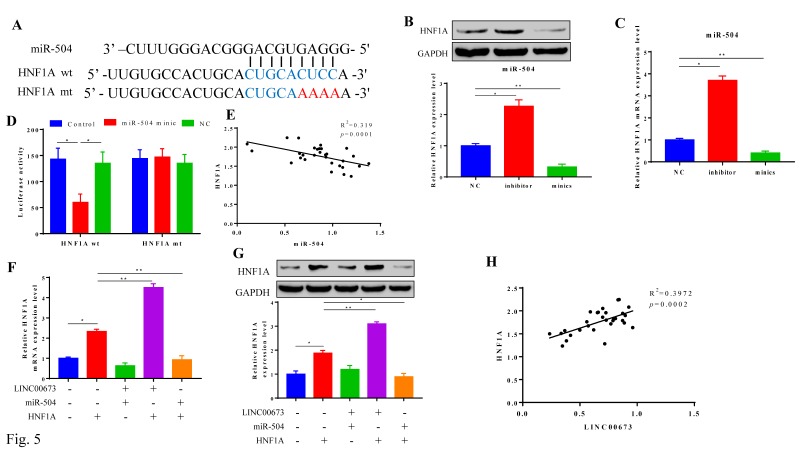
MiR-504 negatively correlated with HNF1A was highly expressed Pancreatic cancer. (A) TargetScan predicted the direct target relationship between miR-504 and HNF1A. (B) Expression HNF1A in miR-504 minics or inhibitors in CanPan-1cells as determined by qRT-PCR. (C) Validation of HNF1A mRNA in transfected with miR-504 minics or inhibitor in CanPan-1 cells tested by qRT-PCR. (D)Luciferase reporter assay. MiR-504 directly targeted at HNF1A. (E)Negative correlation between HNF1A and miR-504 expression in pancreatic cancer patients. (F) Expression HNF1A when miR-504 or LINC00673 exist or not in CanPan-1cells as determined by Western blot. (G) Expression HNF1A mRNA when miR-504 or LINC00673 exist or not in CanPan-1cells as determined by qRT-PCR. (H) Positive correlation between HNF1A and LINC00673 expression in pancreatic cancer patients. *P<0.05, ** P<0.005

HNF1A is a downstream protein in pancreatic cancer, and miR-504 may directly target HNF1A, as predicted by TargetScan. The luciferase reporter assay validated this prediction due to significantly lower luciferase activity in the miR-504 mimic+HNF1A-wt group (Fig. 5A and 5D). The relationship was also confirmed in pancreatic cancer cells by Western blotting (Fig. 5B) and qPCR (Fig. 5C), which again revealed a negative regulatory relationship with miR-504 (Fig. 5E). We also tested the relationship between LINC00673 and HNF1A, as shown in Fig. 5F and 5G, and it was found that miRNA-504 affected the expression of HNF1A and that LINC00673 affected the expression of HNF1A; thus, LINC00673 was positively correlated with HNF1A (Fig. 5H).

### HNF1A suppress pancreatic cancer cells progress

**Figure 6:**
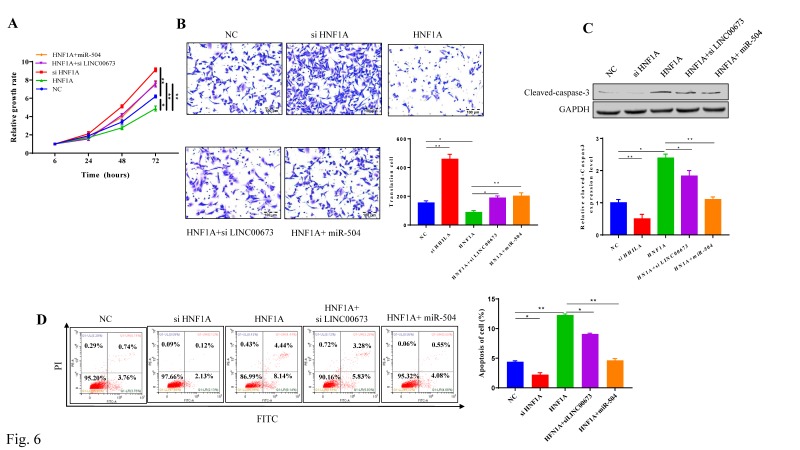
HNF1A suppression inhibited cell progression in pancreatic cancer cells. CCK-8 proliferation assay of specified plasmid or RNA transfection transfected in CanPan-1 cells. (B) Transwell invasion assay in specified plasmid or RNA transfection in CanPan-1 cells. (C) Expression Cleaved-Caspase 3 in specified plasmid or RNA transfected in CanPan-1 cells as determined by western blot. (D)Flow cytometric analysis of apoptosis in specified plasmid or RNA transfected in CanPan-1cells. *P<0.05, ** P<0.01

CCK-8 assays showed that pancreatic cancer cell proliferation was increased in the siHNF1A group, but it was inhibited in the HNF1A-overexpressing group. miR-504 and siLINC00673 both slightly reversed the decrease in expression caused by HNF1A overexpression (Fig. 6A). Cell migration was promoted in the siHNF1A group but suppressed in the HNF1A group, and these effects could be reversed by siLINC00673 and miR-504 mimics (Fig. 6B). The results of the apoptosis assay revealed changes in cell apoptosis that were like the results of the cell migration assay. In the siHNF1A group, the cell apoptosis rate was much lower, but in the HNF1A-overexpressing group, the apoptosis rate was higher, and these effects were reversed by miR-504 and siLINC00673 (Fig. 6D). Consistent with the apoptosis analysis, HNF1A overexpression induced the expression of the pro-apoptotic protein cleaved-caspase3, but siLINC00673 and miR-504 reversed this effect (Fig. 6C).

### LINC 00673 overexpression inhibited caner progression in vivo

**Figure 7:**
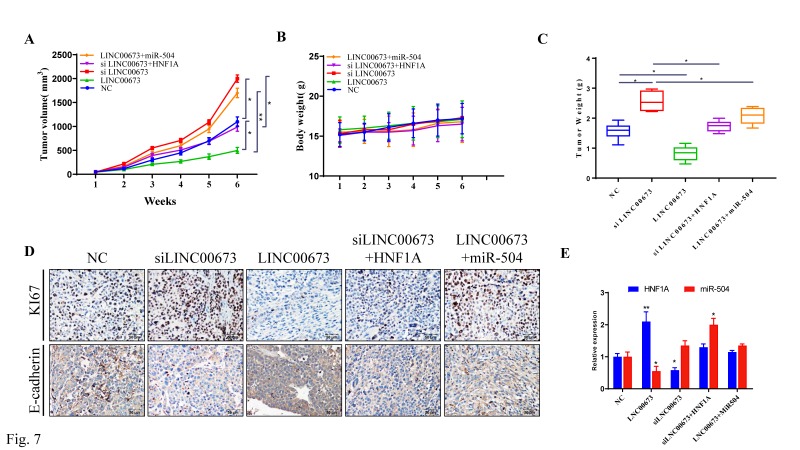
LINC00673 overexpression inhibited caner progression in nude mice. (A) Tumor volume changes after specified plasmid or RNA transfected in CanPan-1cells. (B) Tumor weight changes after specified plasmid or RNA transfected in CanPan-1cells. (C) Body weight changes after specified plasmid or RNA transfected in CanPan-1cells. (D) Immunohistochemical analysis of Ki-67 and E-cadherin derived from xenograft model tumors (magnification, x100). (E) HNF1A and miR-504 expression changes after LINC00673 knockdown or overexpression in Tumors as determined by qRT-PCR. *P<0.05, **P<0.01

As shown in animal experiment, tumor volume (Fig. 7A) and tumor weight (Fig. 7B) were significantly decreased when LINC00673 was overexpressed. However, there is no significant difference in body weight (Fig. 7C). The IHC assay found that protein of proliferation (Ki-67) were promoted in the siLINC00673 group but suppressed in the LINC00673 group, and these effects could be reversed by HNF1A. The expression of migration protein E-cadherin was promoted in the LINC00673 group but suppressed in the siLINC00673 group, and these effects could be reversed by miR-504. (Fig. 7D). HNF1A and miR-504 expression changes were like those observed in vitro after LINC00673 knockdown or overexpression in tumors, as determined by qRT-PCR and shown in Fig. 7E. In addition, we found that LINC00673 may suppress pancreatic cancer deterioration by downregulating miR-504, which restrains HNF1A expression in vivo.

## Discussion

Many studies have shown that the occurrence of invasion and metastasis in pancreatic cancer patients indicates a poor prognosis 15-19. In our study, we used a series of experiments to obtain insight into the biological effects of LINC00673 in pancreatic cancer proliferation and metastasis.

Here, we found that LINC00673 was associated with a good prognosis in pancreatic cancer patients. LINC00673 expression was significantly decreased in primary tumor samples from pancreatic cancer patients compared with adjacent tissues. A lower LINC00673 level in pancreatic cancer patients was correlated with a poor overall survival outcome. In addition, pancreatic cancer expression was significantly decreased in specimens from pancreatic cancer patients who showed metastasis. The expression of LINC00673 was significantly decreased in pancreatic cancer cell lines compared to human pancreatic duct epithelial (HPDE) cells. Overexpression of LINC00673 inhibited apoptosis and suppressed proliferation and migration in pancreatic cancer cells. These findings suggest that LINC00673 functions as a tumor suppressor and that restoration of LINC00673 may be a novel strategy for the treatment of pancreatic cancer proliferation and metastasis.

In this study, we clarified the anti-PC mechanism of LINC00673. The accumulated evidence shows that microRNAs can promote cancer migration and invasion^20^-^22^. We searched for candidate microRNA targets of LINC00673 (mirdb.org) and identified miR-504 as a candidate target of LINC00673. Some studies have shown that miR-504 promotes tumor growth and metastasis in multiple cancers 22-25. Our data show that inhibition of LINC00673 suppressed miR-504 expression and that silencing of miR-504 attenuated the inhibition of migration and invasion by LINC00673. More importantly, our experiments showed that LINC00673 overexpression significantly inhibited the expression of miR-504, suggesting that LINC00673 plays an anticancer role partially through the inhibition of miR-504. In addition, we investigated the mechanism whereby miR-504 regulates migration and invasion in PC. Previous studies have shown that KLK4 can promote the suggesting that HNF-1α might play a tumor suppressor role^26^, ^27^. Here, we used a series of experiments to identify HNF1A as a target gene of miR-504 in PC cells. Our data showed that HNF1A expression was increased or decreased in PC cells by inhibition or ectopic expression of miR-504, respectively. In addition, a luciferase reporter assay showed that miR-504 directly targeted the 3`-UTR of HNF1A. Furthermore, clinical data showed that the expression levels of miR-504 and HNF1A were inversely correlated in PC patient specimens. Additionally, our data demonstrated that restoration of HNF1A blocked the decrease in LINC00673 expression and that miR-504 overexpression induced acceleration of invasion and apoptosis. Taken together, these data suggest that LINC00673 inhibits PC invasion through inhibition of miR-504 by targeting HNF1A.

In summary, we combined clinical and experimental studies to establish the role of LINC00673 in PC metastasis. Overexpression of LINC00673 dramatically inhibits PC apoptosis and invasion. Our findings will be helpful for developing potential therapeutics for PC metastasis.

## Supplementary Material

Supplementary figures and tables.

## Figures and Tables

**Table 1:** The primer sequences information.

Gene	Primer	Sequence
miR-504	3' specific	5'-GACCCTGGTCTGCACTCTATCA-3'
lnc RNA LINC00673	Forward	5′-CCGTGTAAAGAGGCCAGTGT-3′
	Reverse	5′-ACACGAGCCTTCACCATCAG-3′
HNF1A	Forward	5′-AACACCTCAACAAGGGCACTC-3′
	Reverse	5′-CCCCACTTGAAACGGTTCCT-3′
U6	Forward	5′-GCTCGCTTCGGCAGCACAT-3′
	Reverse	5′-AAAATATGGAACGCTTCACG-3′
GAPDH	Forward	5′- CGCTCTCTGCTCCTCCTGTTC-3′
	Reverse	5′- ATCCGTTGACTCCGACCTTCAC-3′
