# Supplementary material for: LNC00673 suppresses proliferation and metastasis of pancreatic cancer via target miR-504/ HNF1A
Source: J Cancer. 2020 Jan 1;11(4):940–8. doi: 10.7150/jca.32855 (PMC6959011; doi:10.7150/jca.32855)
Supplement: Supplementary file 1 — Supplementary figures and tables. [file jcav11p0940s1.pdf]

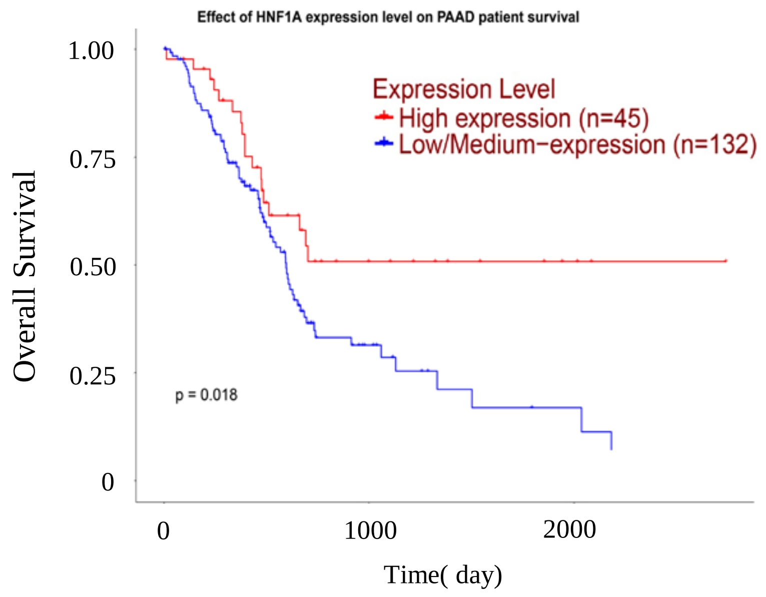

Supplement Figure 1. (A)Association of HNF1A expression with overall survival of pancreatic cancer patients, the data was come from TGGA.

Supplement Figure 1
